# Up-regulation of MIAT aggravates the atherosclerotic damage in atherosclerosis mice through the activation of PI3K/Akt signaling pathway

**DOI:** 10.1080/10717544.2019.1628116

**Published:** 2019-06-25

**Authors:** Guoqiang Sun, Yubo Li, Zhiyong Ji

**Affiliations:** a Department of Cardiology, The First Hospital of Jilin University, Changchun, PR China;; b Department of Pediatrics, Medical College, Changchun, PR China;; c Department of Critical Care Medicine, The First Hospital of Jilin University, Changchun, PR China

**Keywords:** LncRNA MIAT, atherosclerotic damage, atherosclerosis, PI3K/Akt signaling pathway

## Abstract

This study is performed to elucidate the role of long non-coding RNA myocardial infarction associated transcript (lncRNA MIAT) in vulnerable plaque formation in rats with atherosclerosis (AS) through the regulation of the PI3K/Akt signaling pathway. The mice model of AS was established, and the successful modeled AS mice were treated with overexpressed MIAT and silenced MIAT. The levels of blood lipids, atherosclerotic plaques (AP) formation, the lipid content, collagen content, apoptosis of aortic cells, angiogenesis as well as the expression of inflammatory factors, such as tumor necrosis factor α (TNF-α), interleukin-1β (IL-1β), and interleukin-6 (IL-6) were determined through a series of experiments. MIAT was found to be upregulated in AS. Additionally, MIAT up-regulated the levels of blood lipids, promoted AP formation, increased the lipid content and decreased the collagen content of AP, promoted the apoptosis of aortic cells in AS mice by activating the PI3K/Akt signaling pathway. Meanwhile, MIAT was determined to promote angiogenesis as well as the expression of inflammatory factors (IL-1β, IL-6, and TNF-α) in AS mice through the activation of the PI3K/Akt signaling pathway. Furthermore, MIAT activated the PI3K/Akt signaling pathway to participate in AS progression. Our study suggests that upregulation of MIAT can aggravate AS injury in AS mice via the activation of the PI3K/Akt signaling pathway, which could provide a novel target for the treatment of AS.

## Introduction

Atherosclerosis (AS) is one of the most important causes of death in the world, which is a kind of vulnerable cardiovascular disease induced by many risk factors (Pan, 2017). AS is activated by dysfunction of vascular smooth muscle cells (VSMCs) and endothelial cells (ECs), as well as proinflammatory cytokines produced by macrophages (Leischik et al., 2015; Nus & Mallat, 2016). AS is a clinical symptom that contributes to the narrowing of the interior of an artery because of plaque accumulation (Yao et al., 2018). Recent studies have demonstrated that long non-coding RNA (lncRNA) exerted it function in the modulation of AS (Cao et al., 2014; Zhou et al., 2016), but the function and the inner mechanism of lncRNAs in AS remained uncovered. In recent years, the circulating levels of several lncRNAs, such as LincP21 and ANRIL, are markedly elevated in AS, which may be essential in the pathogenesis of AS (Harismendy et al., 2011; Holdt et al., 2013; Bai et al., 2014). Therefore, understanding the role of lncRNAs in AS may provide novel and effective therapeutic methods for AS.

Accumulating data have suggested that lncRNAs exert effects on a great deal of biological processes, including cell differentiation, chromatin remodeling, as well as carcinogenesis (Gibb et al., 2011). Meanwhile, the dysregulation of lncRNAs is associated with tumor proliferation, invasion, and metastasis of several kinds of cancer (Wang et al., 2016; Chen et al., 2017; Lu et al., 2017; Sun et al., 2017). Myocardial infarction-associated transcript (MIAT) is known as a lncRNA which is expressed in heart and fetal brain tissue (Zhu, 2016). Functionally, the overexpression of MIAT is able to result in microvascular dysfunction through promoting ECs proliferation and migration (Yan et al., 2015). MIAT is also capable of affecting cellular functions, including proliferation, apoptosis, and invasion of tumor cells in various human diseases (Sun et al., 2018). A previous study has suggested that MIAT is overexpressed in Ang II-induced cardiac hypertrophy, and the downregulation of MIAT is able to decrease Ang II-induced overexpression of hypertrophic markers, and also alleviate Ang II-induced hypertrophic phenotypes in cells (Zhu, 2016). LncRNAs are capable of epigenetically modulating gene expression in both transcription and post-transcription level, which is participated in multiple signaling pathways (Rinn & Chang, 2012). Luo *et al.* have proposed that the activated phosphoinositide 3-kinase (PI3K)/Protein kinase B (Akt) signaling pathway is able to reduce the levels of reactive oxygen species and lipid deposits which could suppress plaques formation to reverse the progress of AS (Luo et al., 2015). Based on which, we conducted this present study to figure out the role of MIAT in vulnerable plaque formation in rats with AS through the regulation of the PI3K/Akt signaling pathway.

## Materials and methods

### Ethics statement

The study was approved by the animal ethics committee in the First Hospital of Jilin University. All animal work was conducted to relieve their pain according to relevant national and international guidelines.

### Experimental animals and grouping

A total of 50 ApoE (−/−) male mice in clear grade (aging 8 week) were purchased from Beijing Vital River Laboratory Animal Technology Co., Ltd. (Beijing, China). The rats had free access to eating (regular feed or high-fat feed: casein, methionine, sucrose, corn starch, dextrin, anhydrous cream, corn oil, cholesterol, cellulose, minerals, vitamins and antioxidants) and drinking in a standard squirrel cage with 12 h day/night cycle, temperature of 22–25 °C, and humidity of 50-70% as well as high-pressure disinfection, drinking water bottle and padding. Calorie: 4.5 kcal/g, calorie composition: fat 42%, protein 15%, carbohydrate 43%, and cholesterol 0.2%.

The mice were fed with normal diet for 1 week to observe the changes of their body weights and general conditions. The mice were allocated into four groups (*n* = 10): normal group: with free access to regular feed; AS group: with free access to high fat feed; AS + scramble group: with free access to high fat feed and injection of no-loaded adenovirus (5 × 10^9^ PFU/mice) via internal jugular vein at 10 weeks; AS + overexpression (OE)-MIAT group: with free access to high fat feed and injection of MIAT overexpression adenovirus (5 × 10^9^ PFU/mice) via internal jugular vein at 10 weeks; AS + sh-MIAT group: with free access to high fat feed and injection of MIAT silencing adenovirus (5 × 10^9^ PFU/mice) via internal jugular vein at 10 weeks; AS + LY294002 (PI3K/Akt signaling pathway inhibitor): with free access to high fat feed and injection of LY294002 (3 mg/kg, 2 times a week, 2 weeks of injection; Sigma Aldrich (St. Louis, MO, USA)) via peritoneal injection at 10 weeks (Dai et al., 2016). After 12 weeks, the mice in each group were euthanized to obtain blood and specimens.

### Detection of blood lipids and inflammatory factors

Blood was taken from eyeball, and then the serum was separated. The centrifuge tube was placed at room temperature for 2 h, then placed in a refrigerator at 4 °C for 4 h. After blood coagulated into blood clot, it was centrifuged at 4000 rpm for 10 min, and then removed in a clean centrifuge tube and stored at −80 °C. The concentrations of serum total cholesterol (TC), high density lipoprotein cholesterol (HDL-C), low density lipoprotein cholesterol (LDL-C), and triglyceride (TG) were determined by an automatic biochemical analyzer. TC and TG were determined by enzymatic method, LDL and HDL were determined by immunoturbidimetric method. The expression levels of inflammatory cytokines, such as tumor necrosis factor α (TNF-α), interleukin-1β (IL-1β), and interleukin-6 (IL-6) (Nanjing Jiancheng Institute of Bioengineering, Nanjing, Jiangsu, China) were detected by enzyme-linked immunosorbent assay (ELISA). The specific steps were: first, the standard curve was established, then 100 μL serum was added to each well, 2 sub-wells were set up for each well, and the samples were incubated in a 37 °C incubator for 2 h. After the reaction, the plate was washed 5 times and then dried. Next, the primary antibody was supplemented (50 μL/well) and incubated in a 37 °C incubator for 1 h. And then, enzyme-labeled antibody was added (100 μL/well) and incubated in a 37 °C incubator for 1 h. Subsequently, the light-emitting substrate was supplemented (100 μL/well) and incubated in a 37 °C incubator and reacted for 5–10 min. Each well was mixed with 50 μL termination solution, and the optical density (OD) value of each well was measured and recorded on a microplate reader. Finally, the standard curve was drawn to calculate the concentration of TNF-α, IL-1β, and IL-6 in serum.

### Sampling

All the mice were euthanized, the limbs were fixed on the mouse board, the sternum was cut off, and the heart was fixed with tweezers. After cutting off the auricula dextra, the syringe needle was inserted into the apical part about 1–2 mm and then perfused with phosphate buffer solution (PBS). The chest rib and lung tissues were cut off, the esophagus and trachea were cut off from the proximal end, and the heart and aorta were removed. The aortic arch and root of ascending aorta were cut off and soaked in polyformic acid overnight for preparing slices. The skin of the neck of the mouse was cut off and the thyroid gland of the two lobes was separated. In the anterior triangle of both sides of the neck, the muscle fibers of sternocleidomastoid muscle were opened, and the common carotid artery, which was slightly transparent and white, could be seen. The common carotid artery was rinsed with PBS and immersed in 4% paraformaldehyde overnight for preparing slices. The remaining heart and aorta were stored in cryopreserved tubes at −80 °C for tissue extraction of RNA and protein.

### Hematoxylin–eosin (HE) staining

After paraffin embedding, 4-μm thickness slice was made by a histotome. The slices were dewaxed, hydrated with gradient alcohol, stained with hematoxylin solution for 15 min, counterstained with 0.5% eosin solution for 5 min, dehydrated with gradient alcohol, cleared, and sealed. After staining, the nucleus was stained purple and blue by hematoxylin, most of the cytoplasm and non-cellular components were stained pink by eosin, and the fat of the cytoplasm was dissolved in vacuolation.

### Oil red O staining

The OCT-embedded block was sliced into the thickness of 6 μm and stored at −20 °C. The slices were dried for 60–120 min before use, stained with 15 mL oil red O solution, and added with 10–25 mL distilled water. Frozen slices were dried and stained, rinsed with distilled water at 37 °C for 30 s, washed with tap water for 30 s, stained with hematoxylin for 1–3 min, rinsed with water for 30 s, differentiated with 1% hydrochloric acid alcohol for 5 s, and rinsed with tap water for 5 min. The dyeing situation was observed under a microscope. An irregular plaque was observed in the blood vessels by oil red O staining, which was scattered in bright orange-red particles, which were stained lipid droplets in the blood vessels.

### Masson staining

The OCT-embedded block was sliced into the thickness of 6 μm and stored at −20 °C. The slices were dried for 60–120 min before use, stained with Masson compound dye solution for 15 min, washed with 0.2% glacial acetic acid aqueous solution for 5–10 s, and dyed with 0.1% phosphato-tungstic acid for 5–10 min. The dyeing was controlled under the microscope. Next, the slices were washed with 0.2% acetic acid 2 times, 1 min each time, and stained with the bright green dye solution for about 1–2 s, and the dyeing was controlled under the microscope. Subsequently, the slices were washed with 0.2% acetic acid 2 times, 1 min each time, dehydrated with gradient alcohol, immersed in environmental transparent agent for 5 min and sealed automatically by an automatic sealing machine. Masson staining showed irregular atherosclerotic plaques in blood vessels, collagen fibers were green inside and outside the vessel wall and plaque, and smooth muscle cells were red.

### Terminal deoxynucleotidyl transferasec (TdT)-mediated dUTP nick end labeling (TUNEL) staining

After routine paraffin embedding, the tissues were sliced into 4 μm thickness, and dehydrated with gradient ethanol. The slices of aortic tissues were immersed into 0.1 mol/L sodium citrate (200 mL), which were taken out immediately and poured into distilled water. After natural cooling, the slices were rinsed with PBS three times, and then supplemented with the TUNEL reaction mixture and the sealing membrane in a dark humidity box at 37 °C. Afterwards, the samples were supplemented with converter-peroxidase (POD) and the sealing membrane in a dark humidity box at 37 °C. Next, the samples were added with diaminobenzidine (DAB) substrate, counterstained with hematoxylin, rinsed by flowing water, dehydrated by gradient ethanol, cleared by xylene, and sealed by neutral balsam. Those with brown granules in the nucleus were identified as positive, and the number of apoptotic cells was counted.

### Immunohistochemical staining

After routine paraffin embedding, the tissues were sliced into 4 μm thickness, followed by gradient alcohol dehydration, antigenic repair with 100 μL 0.2 mg/mL protein kinase K solution at room temperature for 10 min. After that, the tissues were added with 3% peroxidase blocking solution and incubated at room temperature for 10 min. Next, the tissues were supplemented with 50 μL non-immune sheep serum and incubated at room temperature for 30 min, then added with the anti-rabbit polyclonal antibody vascular endothelial growth factor (VEGF) and CD31, and incubated overnight at 4 °C. The negative control (NC) was replaced by antibody diluent, and then the secondary antibody, goat anti-rabbit IgG (ab6721, 1:1000, Abcam, Cambridge, MA) was supplemented and incubated at room temperature for 30 min. After that, the tissues were supplemented with DAB for coloration, counterstained with hematoxylin and sealed. Five fields of vision were randomly selected for each slice. The cell count of the region was carried out by a computerized medical image analysis system (CMIAS). The experiment was repeated three times.

### Reverse transcription quantitative polymerase reaction chain reaction (RT-qPCR)

Trizol method (Invitrogen, Carlsbad, CA) was used to extract total RNA from aortic tissues. The concentration and purity of extracted RNA were determined. The primers were synthesized by Invitrogen (Carlsbad, CA) (Table 1). Glyceraldehyde phosphate dehydrogenase (GAPDH) was used as an internal control. △Ct = CT (target gene) − CT(internal control), △△Ct = △Ct (experimental group) − △Ct (control group). The relative expression was calculated based on 2^-△△Ct^. Experiments were conducted for three times.

**Table 1.** Primer sequence.

Gene	Sequence
MIAT	F: 5′-AGAGAGGACATGAGGACCCC-3′
	R: 5′-CCTACCTCACAGGGCTGTTG -3′
VEGF	F: 5′-GCTTCGGCAGCACATATACTAAAAT -3′
	R: 5′-CGCTTCACGAATTTGCGTGTCAT -3′
IL-1	F: 5′-TGTGATGAAAGACGGCACAC -3′
	R: 5′-CTTCTTCTTTGGGTATTGTTTGG -3′
IL-6	F: 5′-TGGAGTCACAGAAGGAGTGGCTAAG -3′
	R: 5′-TCTGACCACAGTGAGGAATGTCCAC -3′
TNF-a	F: 5′-CCGATGGGTTGTACCTTGTC -3′
	R: 5′-GGGCTGGGTAGAGAATGGAT -3′
GAPDH	F: 5′-CGACT-TCAACAGCAACTCCCACTCTTCC-3′
	R: 5′-TGGGTGGTCCAGGGTTTCTTACTCCTT-3′

*Note*: F: forward; R: reverse.

### Western blot analysis

The total protein of aortic tissues in each group was extracted. The protein concentration was determined according to the bicinchoninic acid protein assay kit (Wuhan Boster Biological Technology Co., Ltd., Wuhan, Hubei, China). The extracted protein added with uploading buffer and separated with 10% polyacrylamide gel electrophoresis (Wuhan Boster Biological Technology Co., Ltd., Wuhan, Hubei, China). The proteins were transferred onto polyvinylidene fluoride membranes. The membranes were blocked with 5% bovine serum albumin (BSA) for 1 h. Primary antibodies of Caspase-3, Bax, Bcl-2, VEGF, PI3K, p-PI3K, AKT, p-AKT and β-actin (1: 3000, Abcam, Cambridge, MA) were added and incubated at 4 °C overnight, followed by washing three times (5 min per wash) with Tris-buffered saline with Tween 20 (TBST). Corresponding secondary antibodies (Shanghai Miaotong Biotechnology Co., Ltd., Shanghai, China) were added and incubated for 1 h. The membranes were washed for three times with 5 min for each time. Chemiluminescence reagents were employed to develop images. β-Actin was considered as an internal reference. The images of the gels were captured in a Bio-Rad Gel Doc EZ Imager (Bio-Rad, Hercules, CA). The gray values of target protein bands were analyzed by ImageJ software (National Institutes of Health, Bethesda, MA). The experiment was conducted in triplicate.

### Statistical analysis

SPSS 21.0 software (IBM-SPSS Corp, Armonk, NY) was used for analysis. The Kolmogorov–Smirnov test verified that the data were in normal distribution. Results were expressed as mean ± standard deviation, the *t*-test was used in the comparison between two groups, and one-way analysis of variance (ANOVA) was used in the comparison among multiple groups. The Fisher's least significant difference *t* test (LSD-t) was used for pairwise comparison after ANOVA analysis. When *p* value was less than .05, the statistical significance was supposed.

## Results

### Expression of MIAT is increased in aortic tissues of AS rats

**Figure 1.**
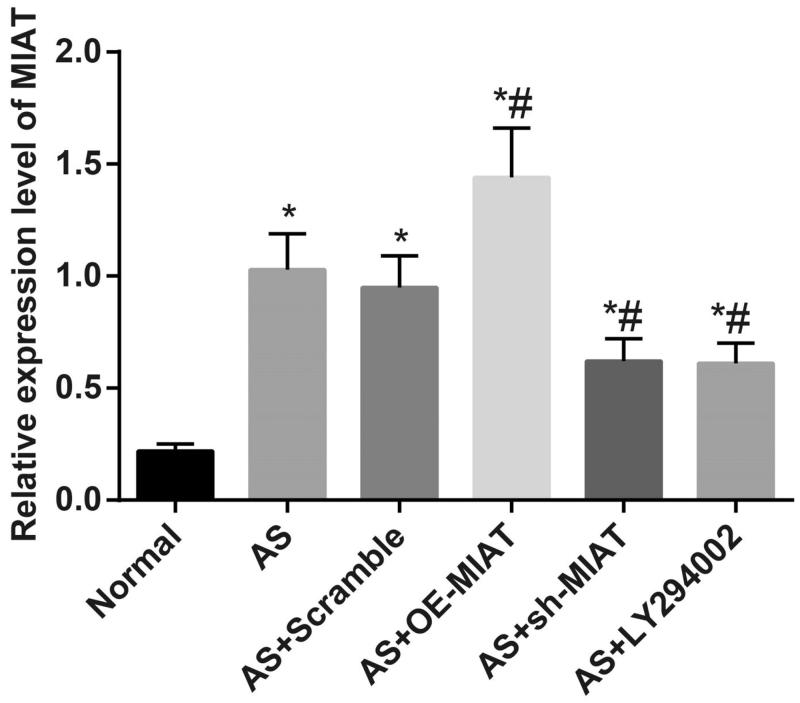
Expression of MIAT in aortic tissues of mice in each group. *N* = 5. *p < .05 versus the normal group; # *p* < .05 versus the AS group. One-way ANOVA was used for data analysis, and LSD-t test was used for comparison after ANOVA analysis.

The results of RT-qPCR (Figure 1) suggested that compared with the normal group, the expression of MIAT was increased in aortic tissues of rats in the other groups (all *p* < .05). There was no significant difference in MIAT expression among the AS group, the AS + scramble group and the AS + LY294002 group (all *p* > .05), but the expression of MIAT was significantly higher in the AS + OE-MIAT group while lower in the AS + sh-MIAT group relative to the AS group (both *p* < .05). The results suggest the successful intervention of MIAT, and MIAT may be involved in the occurrence of AS.

### MIAT up-regulates the levels of blood lipids in AS mice by activating the PI3K/Akt signaling pathway

Automatic biochemical analysis was used to detect the levels of blood lipids (TC, TG, HDL-C, and LDL-C) (Table 2). The results suggested that compared with the normal group, the contents of TC, TG, and LDL-C significantly increased and the content of HDL-C decreased significantly in other groups (all *p* < .05). No evident difference in the serum contents of TC, TG, HDL-C and LDL-C between the AS group and the AS + scramble group (all *p* > .05). In comparison to the AS group, the contents of TC, TG and LDL-C in the AS + OE-MIAT group was significantly increased and the content of HDL-C was significantly decreased; while the AS + sh-MIAT group and the AS + LY294002 group exhibited the reverse trend (all *p* < .05). The results suggest that MIAT may up-regulate the levels of blood lipids in AS mice by activating the PI3K/Akt signaling pathway.

**Table 2.** Changes of blood lipid level in atherosclerotic mice.

Group	TC	TG	LDL-C	HDL-C
Normal	3.55 ± 0.45	0.48 ± 0.04	2.60 ± 0.12	3.22 ± 0.45*
AS	12.06 ± 1.38*	2.18 ± 0.42*	10.21 ± 0.54*	1.67 ± 0.22*
AS + scramble	12.34 ± 1.26*	2.22 ± 0.31*	11.16 ± 0.67*	1.62 ± 0.20*
AS + OE-MIAT	20.28 ± 2.11*^#^	3.04 ± 0.64*^#^	19.67 ± 1.18*^#^	1.04 ± 0.19*^#^
AS + sh-MIAT	7.30 ± 0.99*^#^	1.43 ± 0.27*^#^	6.56 ± 0.14*^#^	2.36 ± 0.31*^#^
AS + LY294002	6.90 ± 1.34*^#^	1.38 ± 0.25*^#^	6.91 ± 0.17*^#^	2.30 ± 0.27*^#^

*Note*: *N* = 5. One-way ANOVA was used for data analysis, and LSD-t test was used for comparison after ANOVA.

*p < .05 versus the normal group.

# *p* < .05 versus the AS group.

### MIAT promotes atherosclerotic plaques (AP) formation, increases the lipid content of AP and decreases the collagen content of AP in AS mice by activating the PI3K/Akt signaling pathway

**Figure 2.**
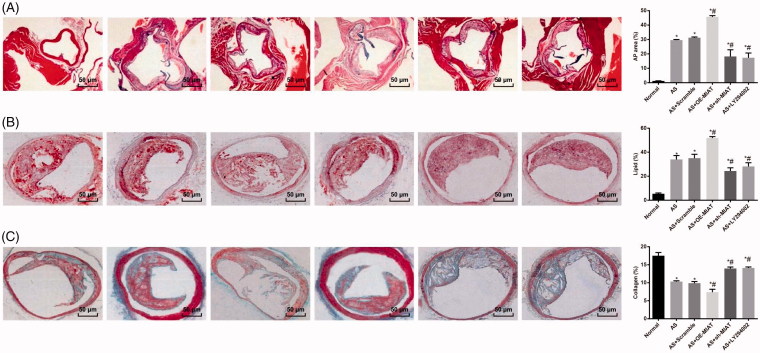
MIAT promotes AP formation, increases the lipid content and decreases the collagen content of AP in AS mice by activating the PI3K/Akt signaling pathway. (A) AP formation in aorta of mice in each group was observed by HE staining (× 200). (B) Lipid content in aorta of mice in each group was observed by oil red O staining (× 200). (C) Content of collagen in aorta of mice in each group was measured by Masson staining (× 200). *N* = 5. One-way ANOVA was used for data analysis, and LSD-t test was used for comparison after ANOVA. * *p* < .05 versus the normal group; # *p* < .05 versus the AS group.

As shown in Figure 2(A), the results of HE staining indicated that in mice of the normal group, the integrity of the intima was partially destroyed, and a small number of foam cells and inflammatory cells were accumulated in the endometrium. In mice of the AS group, endometrial integrity was broken, foam cells and inflammatory cells were gathered, and local AP were formed. In mice of the AS + OE-MIAT group, it could be seen that the integrity of the intima was destroyed, a large number of foam cells and inflammatory cells were found in the subintimal membrane of the mice. Plaque rupture was observed in some of the AP. Compared with the normal group, the area of aortic AP in other groups increased significantly (all *p* < .05). There was no significant difference in the area of aortic AP between the AS group and the AS + scramble group (*p* > .05), but the area of aortic AP in the AS + OE-MIAT group was significantly higher while which was significantly lower in the AS + sh-MIAT group and the AS + LY294002 group in comparison to that in the AS group (all *p* < .05). The results suggest that MIAT may promote AP formation in AS mice by activating the PI3K/Akt signaling pathway.

The results of oil red O staining suggested that relative to the normal group, the lipid content in the area of aortic AP in other groups increased significantly (all *p* < .05). There was no significant difference in lipid content in the area of aortic AP between the AS group and the AS + scramble group (*p* > .05), but the lipid content in the area of aortic AP in the AS + OE-MIAT group was significantly higher while which was significantly lower in the AS + sh-MIAT group and the AS + LY294002 group relative to that in the AS group (all *p* < .05). The results suggest that MIAT may increase the lipid content of AP in AS mice by activating the PI3K/Akt signaling pathway.

The results of Masson staining showed that the collagen content in the area of aortic AP in other groups decreased significantly relative to the normal group (all *p* < .05). No obvious difference was detected in collagen content in the area of aortic AP between the AS group and the AS + scramble group (*p*> .05). The collagen content in the area of aortic AP in the AS + OE-MIAT group was significantly lower while which was significantly higher in the AS + sh-MIAT group and the AS + LY294002 group than that in the AS group (all *p* < .05). The results suggest that MIAT may decrease the collagen content of AP in AS mice by activating the PI3K/Akt signaling pathway.

### MIAT promotes the apoptosis of aortic cells in AS mice by activating the PI3K/Akt signaling pathway

**Figure 3.**
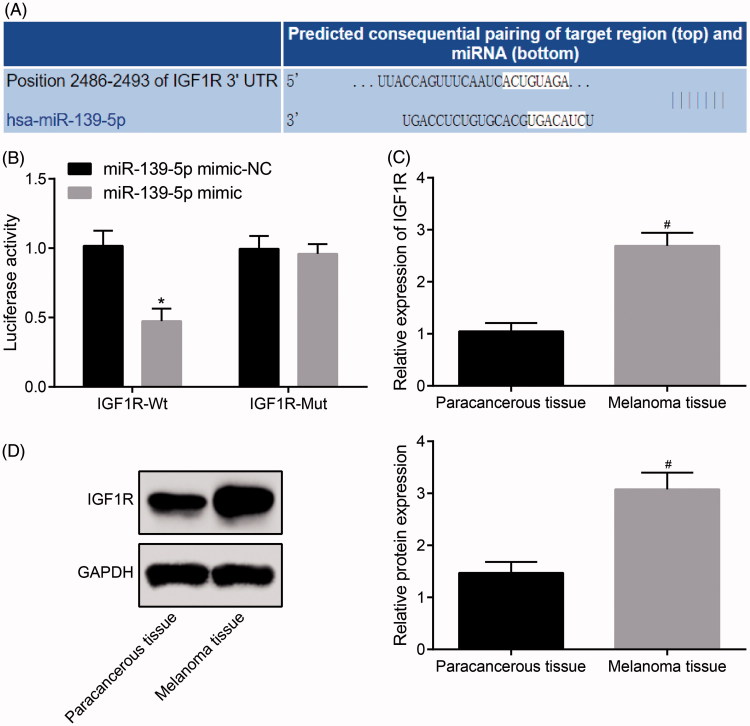
MIAT promotes the apoptosis of aortic cells in AS mice by activating the PI3K/Akt signaling pathway. (A) Detection of apoptosis in mouse aortic cells by TUNEL staining (× 200). (B) Detection of apoptosis-related protein expression in mouse aorta by western blot analysis. *N* = 5. One-way ANOVA was used for data analysis, and LSD-t test was used for comparison after ANOVA. * *p* < .05 versus the normal group; # *p* < .05 versus the AS group.

As shown in Figure 3(A), the results of TUNEL staining indicated that compared with the normal group, the apoptosis of aortic cells in other groups was significantly increased (all *p* < .05). In contrast to the AS group, the apoptosis of aortic cells in the AS + scramble group was not significantly different (*p* > .05), while which was significantly increased in the AS + OE-MIAT group and decreased in the AS + sh-MIAT and the AS LY294002 groups (all *p* < .05).

The results of western blot analysis (Figure 3(B)) suggested that the expression of caspase-3 and Bax in aortic tissues of mice fed with high fat in other groups was significantly higher and the expression of Bcl-2 was significantly lower than that in the normal group (all *p* < .05). There was no significant difference in the expression of apoptosis-related indexes between the AS + scramble group and the AS group (all *p* > .05). The expression of caspase-3 and Bax in the aortic tissues of mice was significantly up-regulated and the expression of Bcl-2 was down-regulated in the AS + OE-MIAT group in contrast to the AS group (all *p* < .05). The AS + sh-MIAT and the AS + LY294002 groups showed an opposite trend. To conclude, MIAT promotes the apoptosis of aortic cells in AS mice by activating the PI3K/Akt signaling pathway.

### MIAT promotes angiogenesis in AS mice by activating the PI3K/Akt signaling pathway

**Figure 4.**
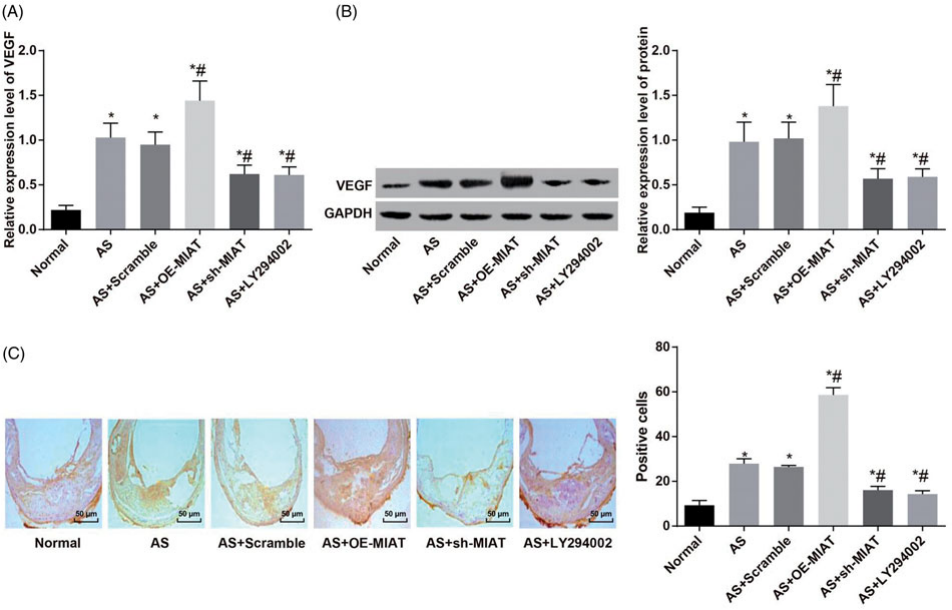
MIAT promotes angiogenesis in AS mice by activating the PI3K/Akt signaling pathway. (A) Detection of VEGF mRNA expression in aorta of mice by RT-qPCR. (B) Detection of VEGF protein expression in mouse aorta by western blot analysis. (C) Expression of VEGF protein in aorta of mice detected by immunohistochemical staining (× 200). *N* = 5. One-way ANOVA was used for data analysis, and LSD-t test was used for comparison after ANOVA. * *p* < .05 versus the normal group; # *p* < .05 versus the AS group.

According to the results of RT-qPCR and western blot analysis (Figure 4(A,B)), we found that compared with the normal group, the mRNA and protein expression of VEGF in the aortic tissues of mice in other groups were significantly up-regulated (all *p* < .05). There was no significant difference in the mRNA and protein expression of VEGF in the aortic tissues of mice between the AS + scramble group and the AS group (*p* > .05). Relative to the AS group, the mRNA and protein expression of VEGF in the aortic tissues of mice in the AS + OE-MIAT group was significantly up-regulated, while which was significantly decreased in the AS + sh-MIAT group and the AS + LY294002 group (all *p* < .05).

The results of immunohistochemical staining (Figure 4(C)) indicated that there was no positive expression of VEGF in aortic tissues of mice in the normal group, but there was significant expression in other groups of AS mice fed with high fat. The positive expression of VEGF in other groups was significantly higher than in the normal group (*p* < .05). There was no significant difference in VEGF expression in aortic tissues between the AS + scramble group and the AS + OE-MIAT group (*p* > .05). The positive expression of VEGF in aortic tissues of mice in the AS + OE-MIAT group was significantly higher while which was significantly decreased in the AS + sh-MIAT group and the AS + LY294002 group (all *p* < .05). The results suggest that MIAT may promote angiogenesis in AS mice by activating the PI3K/Akt signaling pathway.

### MIAT promotes the expression of inflammatory factors in AS mice by activating the PI3K/Akt signaling pathway

**Figure 5.**
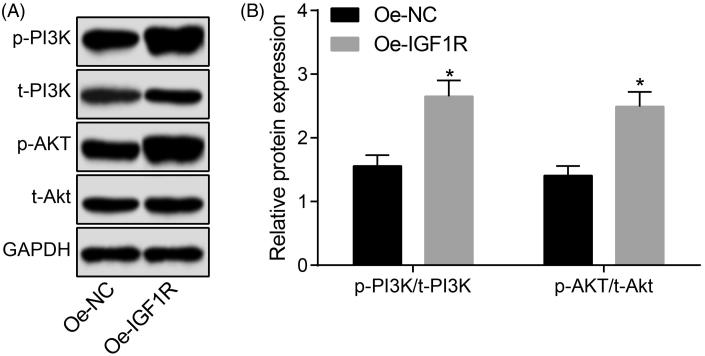
MIAT promotes the expression of inflammatory factors in AS mice by activating the PI3K/Akt signaling pathway. (A) Expression of inflammatory factors (IL-1β, IL-6, and TNF-α) in serum of mice measured by ELISA. (B) Expression of inflammatory factors (IL-1β, IL-6, and TNF-α) in aortic tissues of mice detected by RT-qPCR. *N* = 5. One-way ANOVA was used for data analysis, and LSD-t test was used for comparison after ANOVA. * *p* < .05 versus the normal group; # *p* < .05 versus the AS group.

ELISA and RT-qPCR were used to detect the expression of inflammatory factors (IL-1β, IL-6, and TNF-α) in serum and aortic tissues of mice (Figure 5(A,B)). The expression of IL-1β, IL-6, and TNF-α in serum and aortic tissues of mice in other groups was significantly higher in other groups when compared with those in the normal group (all *p* < .05). There was no significant difference in expression of IL-1β, IL-6, and TNF-α in serum and aortic tissues of mice between the AS + scramble group and the AS group (all *p* > .05). The expression of IL-1β, IL-6, and TNF-α in the AS + OE-MIAT group was significantly higher and expression of which was significantly decreased in the AS + sh-MIAT group and the AS + LY294002 group (all *p* < .05). The results suggest that MIAT may promote the expression of inflammatory factors in AS mice by activating the PI3K/Akt signaling pathway.

### MIAT activates the PI3K/Akt signaling pathway

**Figure 6.**
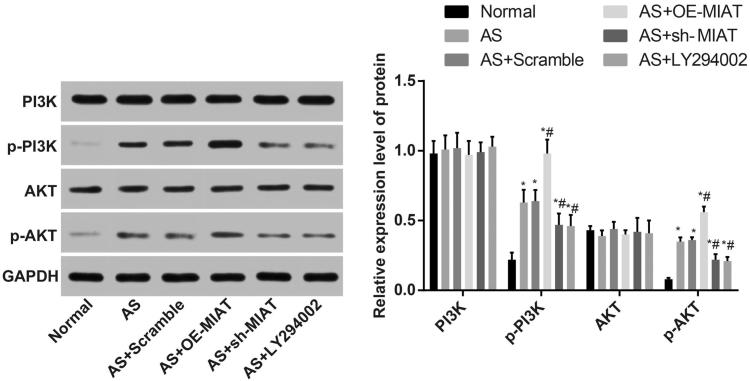
MIAT activates the PI3K/Akt signaling pathway. *N* = 5. One-way ANOVA was used for data analysis, and LSD-t test was used for comparison after ANOVA. * *p* < .05 versus the normal group; # *p* < .05 versus the AS group.

The activation state of the PI3K/Akt signaling pathway was determined by western blot analysis. The results suggested that (Figure 6) there was no significant difference in the expression of PI3K and Akt in the aortic tissues of mice in each group (all *p* > .05). Compared with the normal group, the expression of p-PI3K and p-Akt in the aortic tissues of mice in other groups were significantly increased (all *p* < .05). There was no significant difference in the expression of p-PI3K and p-Akt between the AS + scramble group and the AS group (both *p* > .05). The expression of p-PI3K and p-Akt in the aortic tissues of mice in the AS + OE-MIAT group was significantly higher, and in the AS + sh-MIAT group and the AS + LY294002 group was significantly decreased (all *p* < .05). The results suggest that MIAT can activate the PI3K/Akt signaling pathway.

## Discussion

As one of the most frequent vascular disorders, AS is the root reason of a series of diseases, such as stroke, myocardial infarction, and gangrene (Schober et al., 2014). Recent studies have demonstrated that lncRNAs are associated with the progression of AS, while there is no report concentrated on the effect of MIAT in AS. Thus, there is an urgent need to seek for the effective therapeutic target to decrease the incidence of AS. In this present study, we aim to explore the role of MIAT in vulnerable plaque formation in rats with AS through the regulation of the PI3K/Akt signaling pathway. Collectively, the results of this study suggest that up-regulation of MIAT can aggravate the development of AS in AS mice, and its mechanism is related to the activation of the PI3K/Akt signaling pathway.

One of the most significant findings in this study was that the expression of MIAT was up-regulated in AS mice. Similar to our study, lncRNA HOXC-AS1 was found to be poorly expressed in AS, which suggested that HOXC-AS1 could be consider as a useful biomarker for the treatment of AS (Huang et al., 2016). Additionally, Shan et al. have also found that lncRNA RNCR3 was overexpressed in both mouse and human aortic AS, indicating that RNCR3 could act as a potential target for treating AS (Shan et al., 2016). Another study suggested that MIAT is significantly overexpressed in Ang II-induced cardiac hypertrophy in a mouse model and in H9c2 cells (Zhu, 2016), which is in line with the results in our study. In ischemic stroke patients, MIAT expression was significantly upregulated, and MIAT was a prognostic marker of prognosis in patients with IS (Zhu et al., 2018).

Additionally, our study also found that MIAT promoted angiogenesis as well as the expression of inflammatory factors (IL-1β, IL-6, and TNF-α) in AS mice by activating the PI3K/Akt signaling pathway. PI3K/Akt signaling pathway enables the NF-κB transcription factors to enhance the activity of inflammatory medium gene, thereby contributing to the generation of many cytokines (Kang et al., 2015). IL-6 plays a critical part in cardiovascular disease, which could activate ECs and modulate the extracellular lipids (Hibi et al., 1996). Meanwhile, IL-6 is able to induce the synthesis of matrix metalloproteinases and control their functions for the AS vulnerable plaques (Luckett & Gallucci, 2007; Sundararaj et al., 2009). TNF-α is a significant cytokine implicated in the progress of AS (Ozeren et al., 2003). TNF-α is found to suppress the expression of P4H, which induces the formation of collagen that also contribute to the formation of AS vulnerable plaques (Kang et al., 2015), and it can also suppress the ECs’ function, promote lipid deposition and the production of inflammatory cytokines to aggravate AS (Kleemann et al., 2008; Zhang et al., 2009). It is well known that activated PI3K/Akt signaling pathway is able to promote the aggregation of inflammatory cells to accelerate the development of AS (Morello et al., 2008). Therefore, the inhibition of the PI3K/Akt signaling pathway enables to suppress NF-κB expression to ameliorate the progress of AS (Sousa et al., 2001; Poon et al., 2002; Mourani et al., 2004).

Furthermore, the results of this study also suggested that MIAT activated the PI3K/Akt signaling pathway. As previously reported, downregulated MIAT alleviated microvascular dysfunction *in vivo*, and the change of the specific signaling pathways are implicated in cell proliferation and migration of ECs (Yan et al., 2015). Evidence has shown that lnc00113 is upregulated in serum of patients with AS, and lnc00113 induces proliferation and migration through the activation of the PI3K/Akt/mTOR signaling pathway in AS, which may provide therapeutic methods for AS (Yao et al., 2018). Another report has revealed that the inhibition of the Akt/mTOR signaling pathway suppresses the progression of AS and enhances the stability of AS plaques by activating macrophage autophagy (Zhai et al., 2014).

In summary, our study demonstrates that the up-regulation of MIAT is found in AS mice and up-regulation of MIAT can aggravate the atherosclerotic damage in AS mice through the activation of the PI3K/Akt signaling pathway. However, further study is need for verifying the potential mechanism for lncRNA MIAT for the treatment of AS.
